# Loss of ZG16 is regulated by miR-196a and contributes to stemness and progression of colorectal cancer

**DOI:** 10.18632/oncotarget.13435

**Published:** 2016-11-17

**Authors:** Xiaobing Chen, Peng Du, Junjun She, Liang Cao, Yingchao Li, Hongping Xia

**Affiliations:** ^1^ Department of Oncology, Henan Cancer Hospital, The Affiliated Cancer Hospital of Zhengzhou University, Zhengzhou 450008, China; ^2^ Department of Colorectal Surgery, Xinhua Hospital, Affiliated to Shanghai Jiao Tong University School of Medicine, Shanghai 200092, China; ^3^ Department of General Surgery, The First Affiliated Hospital of Xi’An Jiaotong University, Xi’An 710061, China; ^4^ Department of Gastroenterology, The First Affiliated Hospital of Xi’an Jiaotong University, Xi’an 710061, China

**Keywords:** ZG16, Colorectal cancer, LGR5, stemness, miR-196a

## Abstract

Colorectal cancer (CRC) is one of the most common malignant tumour and the leading cause of cancer-related mortality worldwide. Clarification of the mechanism that underlies CRC tumorigenesis and progression therefore is urgently needed ffor developing novel therapies. Through analysis of The Cancer Genome Atlas (TCGA) dataset, we identified an interesting gene, ZG16, which is significantly decreased in CRC samples compared to adjacent non-tumor tissues and associated with prognosis of patients. We found that the expression of ZG16 correlated with CRC related genes which were regulated by APC/CTNNB1 pathway. Interestingly, the expression of ZG16 was negatively correlated with CRC stem cell marker, LGR5. Overexpression of ZG16 significantly inhibits growth and sphere formation of stem-like CRC cells. Moreover, we also identified an upstream regulator of ZG16, miR-196a, which was significantly overexpressed in CRC and promotes cell growth and stemness. In conclusion, this study demonstrated that loss of ZG16 is regulated by miR-196a and contributes to stemness and progression of CRC, which may provide a promising therapeutic strategy for advanced CRCs.

## INTRODUCTION

Colorectal cancer (CRC) is one of the most common malignant tumour and the leading cause of cancer-related mortality in the world. It is estimated that 134,490 new cases of CRC will be diagnosed and that 49,190 patients will die from this disease during 2016 [1]. The stage I/II colon cancer patients mainly undergo partial or total colectomy, while the stage I rectal cancer patients are mainly treated by proctectomy or proctocolectomy [2]. Chemotherapy is the main treatment for advanced stage CRCs. When CRCs are detected at early stage with a localized disease, the 5-year survival rate is around 90%. However, a large number of patients have been diagnosed at an late stage, whereas the 5-year survival rate is only up to 10% [2].

Comprehensive molecular characterization of human CRCs has been investigated by The Cancer Genome Atlas(TCGA) [3]. The study indicated that the genomic alteration of colon and rectum tumors is quite similar. However, most of genomic alterations in CRCs tumorigenesis and treatment have not been fully clarified [3]. Clarification of the mechanisms that underlie CRC tumorigenesis and progression therefore is urgently needed for developing novel therapies. Further understanding the genomic alteration of CRCs and the involved biological signaling pathways may enable to identify potential therapeutic targets.

Through comprehensive analysis of the TCGA dataset, we identified an interesting gene, ZG16, which is significantly decreased in CRC samples compared to adjacent non-tumor samples. In current study, we validated the expression of ZG16 in a number of paired CRC tissues. We observed that the expression of ZG16 correlated with CRC related genes which were regulated by the APC/CTNNB1 pathway. Interestingly, the expression of ZG16 was negatively correlated with the CRC stem cell marker, LGR5. Overexpression of ZG16 significantly inhibits growth and sphere formation of stem-like CRC cells. Moreover, we also identified an upstream regulator of ZG16, miR-196a, which was significantly overexpressed in CRC and promotes cell growth and stemness of CRC cells. In conclusion, this study demonstrated that loss of ZG16 is regulated by miR-196a overexpression and contributes to stemness and progression of CRC, which may provide a promising therapeutic strategy for advanced CRCs.

## RESULTS

### ZG16 is significantly decreased in CRC and correlated with prognosis

**Figure 1:**
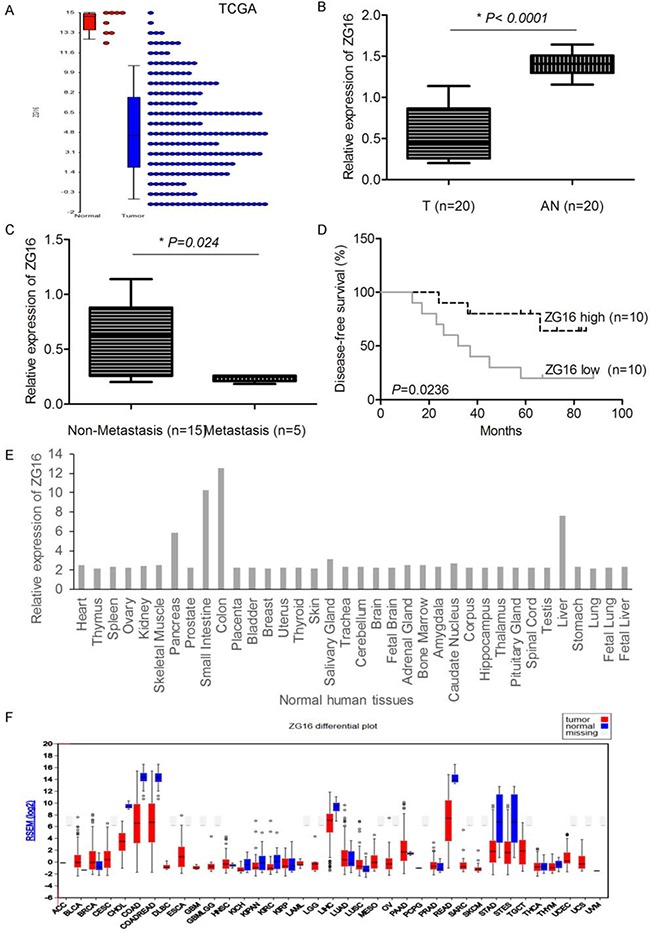
ZG16 is significantly decreased in CRC and correlated with prognosis **A**. The normalized relative expression level of ZG16 in tumor and non-tumor samples in TCGA COAD dataset. **B**. The normalized relative expression of ZG16 in 20 pairs of CRC and adjacent normal samples by RT-qPCR analysis (T: Tumor, AN: Adjacent Normal). **C**. The expression of ZG16 was associated with tumor metastasis of CRC. **D**. The expression of ZG16 was significantly associated with patients’ survival. **E**. The expression of ZG16 was mainly expressed in small intestine, colon and liver of normal human tissue profile analysis of dataset GSE2361. **F**. The expression of ZG16 was significantly downregulated in the TCGA-COAD and rectum adenocarcinoma (READ) of TCGA dataset.

Through analysis of the RNA sequencing data of The Cancer Genome Atlas Colon Adenocarcinoma (TCGA-COAD) data collection, we observed that ZG16 was significantly down-regulated in CRC compared to non-tumor tissue samples (Figure 1A). We further examined twenty pair of CRCs versus non-tumor tissue samples by real time qRT-PCR. This result further confirmed that ZG16 is significantly decreased in CRCs (Figure 1B). Moreover, the expression of ZG16 was down-regulated in metastasis cases compared to non-metastasis cases (Figure 1C). When the median expression level of ZG16 in the CRC samples studied in our analysis was chosen as the cut-off point for ZG16 low and high, low expression of ZG16 in CRC tissues was significantly related with shorter overall survival of CRC patients based on the Fisher's exact test and Kaplan-Meier analysis (Figure 1D). We further analyzed the expression of ZG16 by profiling of 36 types of normal human tissues from dataset GSE2361 [4]. Interestingly, the expression of ZG16 was mainly expressed in small intestine, colon and liver (Figure 1E). However, the expression of ZG16 was significantly down-regulated in the TCGA-COAD and rectum adenocarcinoma (READ) (Figure 1F), suggesting that loss of ZG16 may play an important role in the development of CRCs.

### The expression of ZG16 correlates with CRC related genes which were regulated by the APC/CTNNB1 pathway

**Figure 2:**
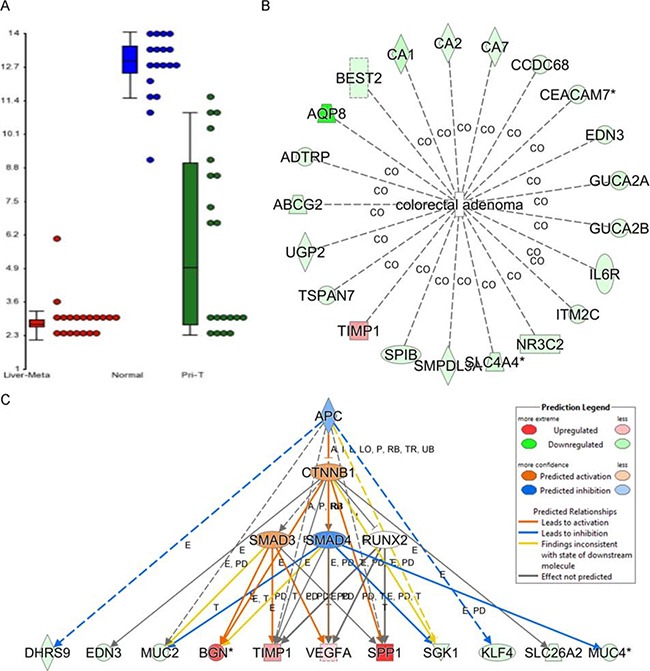
The expression of ZG16 correlates with CRC related genes which were regulated by APC/CTNNB1 pathway **A**. The expression of ZG16 was significantly downregulated in CRC, especially CRC with liver metastasis based on the gene expression profile dataset of GSE49355. **B**. Ingenuity Pathway Analysis (IPA) indicated that the function of a panel of genes which are highly correlated with the expression of ZG16 (r>0.8 using Pearson correlation analysis) are mainly related to CRC. **C**. The IPA analysis indicated that most of these CRC related genes were regulated by APC/CTNNB1 pathway.

To further investigate the expression and role of ZG16 expression in the development of CRC, we examined the data of GSE49355, which including gene expression profile of 57 samples with normal colon, primary tumors and hepatic metastases information. Consistently, the expression of ZG16 was also shown significantly down-regulated in CRCs, especially CRCs with liver metastasis (Figure 2A). To understanding the molecular mechanism of ZG16 in CRC development and metastasis, we further investigated the ZG16 correlated genes. Interestingly, we found that a panel of genes was highly correlated with the expression of ZG16 (r>0.8 using Pearson correlation analysis) (Supplementary Table S1). Ingenuity Pathway Analysis (IPA) indicated that the function of many of these genes was mainly related to CRC (Figure 2B). The IPA result also indicated that most of these CRC related genes were regulated by the APC/CTNNB1 pathway (Figure 2C). It is well known that both APC and CTNNB1 mutation are frequently happened in CRCs and are critical for carcinogenesis and progression of CRCs, including tumor initiation and metastasis [5]. Therefore, loss of ZG16 may also play an important role in CRC initiation and progression.

### Overexpression of ZG16 inhibits growth and sphere formation of stem-like CRC cells

**Figure 3:**
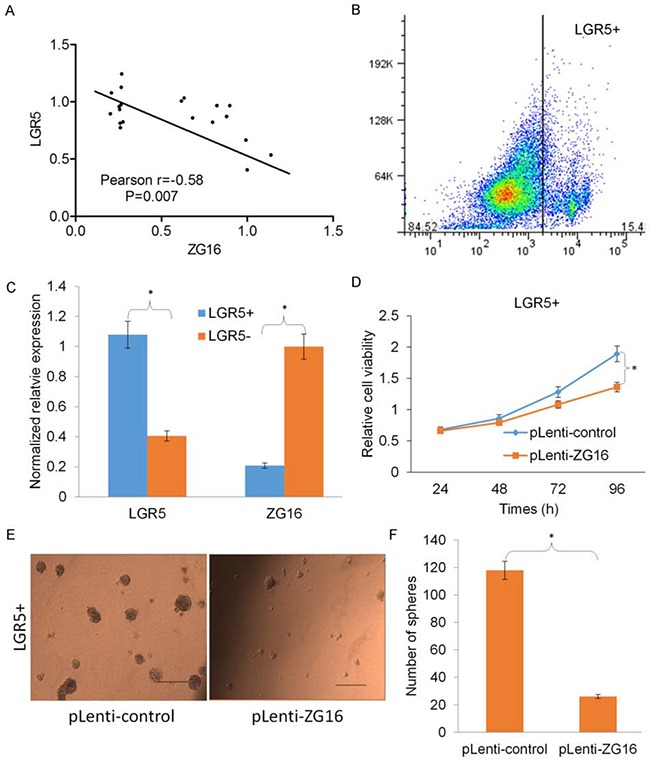
Overexpression of ZG16 inhibits growth and sphere formation of stem-like CRC cells **A**. The loss of ZG16 was highly correlated with overexpression of LGR5. **B**. The isolation of LGR5+ and LGR5- cell population from CRC HT-29 cells by flow cytometry sorting. **C**. The expression of ZG16 and LGR5 was examined in the isolated LGR5+ and LGR- cells by real time qRT-PCR analysis. **D**. The MTS assay result showed the overexpression of ZG16 significantly inhibits LGR5+ CRC cell growth. **E** and **F**. The representative images (E) and quantification analysis (F) of tumour sphere formation assay showed that overexpression of ZG16 also significantly inhibited the sphere forming ability of LGR5+ CRC cells.

To understand the role of ZG16 in CRC initiation and progression, we investigated the correlation of ZG16 with stem-like cell markers of CRC. Interestingly, we observed that the loss of ZG16 was highly correlated with overexpression of LGR5, which has been reported as a marker for stem-like cells in CRC (Figure 3A) [6]. To validate this observation, we further isolated LGR5+ and LGR5- cell population from CRC cells (Figure 3B). Interestingly, the inverse expression of ZG16 and LGR5 was observed by real time qRT-PCR analysis (Figure 3C). We next overexpressed ZG16 by infecting LGR5+ CRC cells with lentivirus mediated ZG16 (pLenti-ZG16). The result showed that overexpression of ZG16 significantly inhibits LGR5+ CRC cell growth (Figure 3D). The tumour sphere formation assay indicated that ZG16 overexpression also significantly inhibited the sphere forming ability of LGR5+ CRC cells (Figure 3E and 3F). These data suggest that overexpression of ZG16 inhibits growth and sphere formation of stem-like CRC cells.

### miR-196a is an upstream regulator of ZG16 in CRC cells

**Figure 4:**
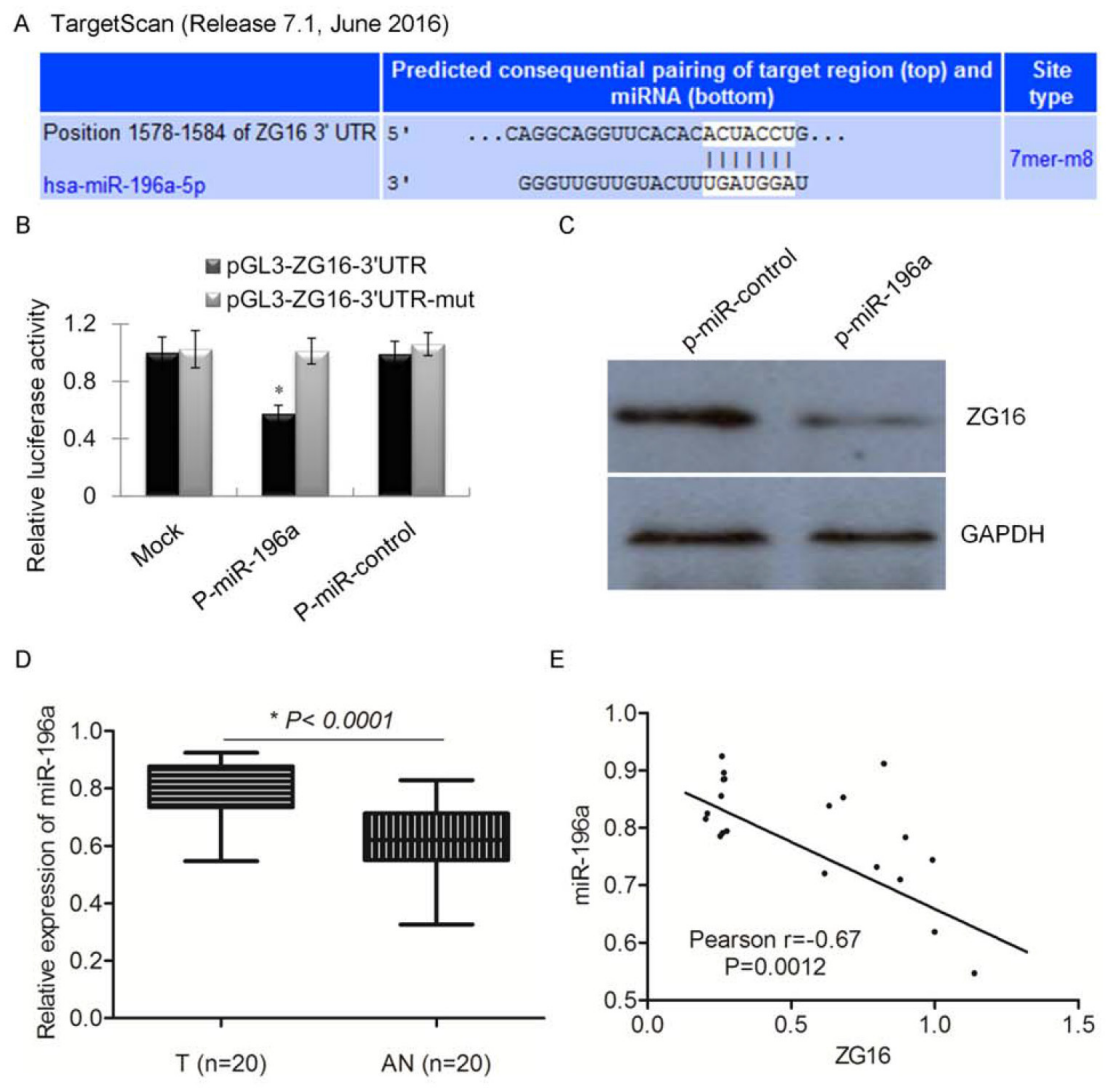
miR-196a is an upstream regulator of ZG16 in CRC cells **A**. The TargetScan prediction indicates that ZG16 is potentially regulated by miR-196 family. **B**. Luciferase activity in cells cotransfected with p-miR-196a and pGL3-ZG16-3’UTR-wt vector was decreased when compared with the control. **C**. Overexpression of miR-196a decreased the expression of ZG16 in LGR5- HT-29 cells. **D**. The expression of miR-196a was detected overexpression in CRC tumor samples compared to adjacent normal tissues by qRT-PCR analysis. **E**. The expression of miR-196a was also reversely correlated with the expression of ZG16 in the CRC samples.

To investigate the potential upstream post-transcriptional regulatory microRNAs of ZG16, we used the TargetScan for prediction. The prediction indicates that ZG16 is potentially regulated by miR-196 family (Figure 4A). To further confirm whether miR-196a could directly bind to the 3’-UTRs of ZG16 mRNA, the predicted binding sites sequence of ZG16-3’UTR or a mutated predicted binding sites sequence was cloned to pGL3to generate pGL3-ZG16-3’UTR-wt or pGL3-ZG16-3’UTR-mut. These constructs were co-transfected with the p-miR-196a or p-miR-control plasmid into LGR5- HT-29 cells. The transfection efficiency was normalized with a co-transfected renilla luciferase vector (pRL-TK). The normalized relative luciferase activity in CRC cells co-transfected with p-miR-196a and pGL3-ZG16-3’UTR-wt vector was inhibited (Figure 4B). Consistently, the protein expression of ZG16 in CRC cells transfection with p-miR-196a and p-miR-control was examined. The data indicated that overexpression of miR-196a significantly decreased the expression of ZG16 in LGR5- HT-29 cells. The expression of miR-196a was also significantly higher in CRC tissue samples compared to adjacent normal tissues (Figure 4D). The expression of miR-196a was also inversely correlated with the expression of ZG16 in the CRC samples (Figure 4E).

### miR-196a promotes tumor cell growth and stemness of CRC cells

**Figure 5:**
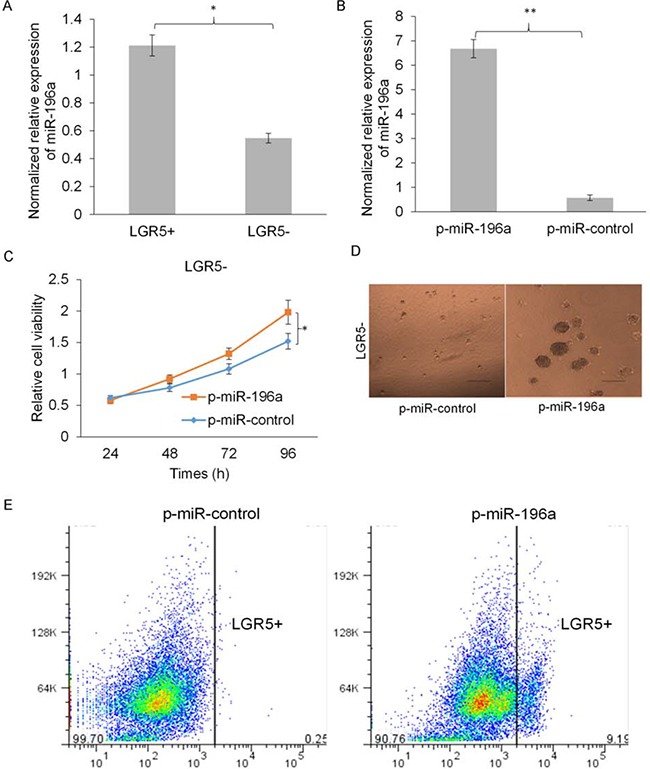
miR-196a promotes cell growth and stemness of CRC cells **A**. The expression of miR-196a was much more increased in LGR5+ than LGR5- CRC cells by qRT-PCR analysis. **B**. The overexpression effect of p-miR-196a transfection was examined by real time qRT-PCR. **C**. Overexpression of miR-196a significantly promotes cell growth of LGR5- CRC cells by MS analysis. **D**. The representative images of tumor spheres showed that overexpression of miR-196a promotes the sphere forming ability of LGR5- CRC cells. **E**. The representative flow cytometry analysis images showed that overexpression of miR-196a increased the LGR+ CRC cell population.

To further investigate the function of miR-196a in CRC tumor initiation, we firstly examined the expression of miR-196a in the isolated LGR5+ and LGR5- CRC cells. Interestingly, miR-196a was much more highly expressed in LGR5+ than LGR5- CRC cells (Figure 5A). We next overexpressed miR-196a in LGR5- CRC cells by lentivirus mediated miR-196a infection (p-miR-196a). The overexpression effect of p-miR-196a was examined by real time qRT-PCR (Figure 5B). Overexpression of miR-196a significantly promotes cell growth of LGR5- CRC cells (Figure 5C). Moreover, overexpression of miR-196a also promotes the sphere forming ability of LGR5- CRC cells and increased the LGR+ CRC cell population (Figure 5D and 5E). These data suggested that overexpression of miR-196a promotes cell growth and stemness of CRC cells.

### miR-196a / ZG16 contributes tumor growth of stem-like CRCs *in vivo*

**Figure 6:**
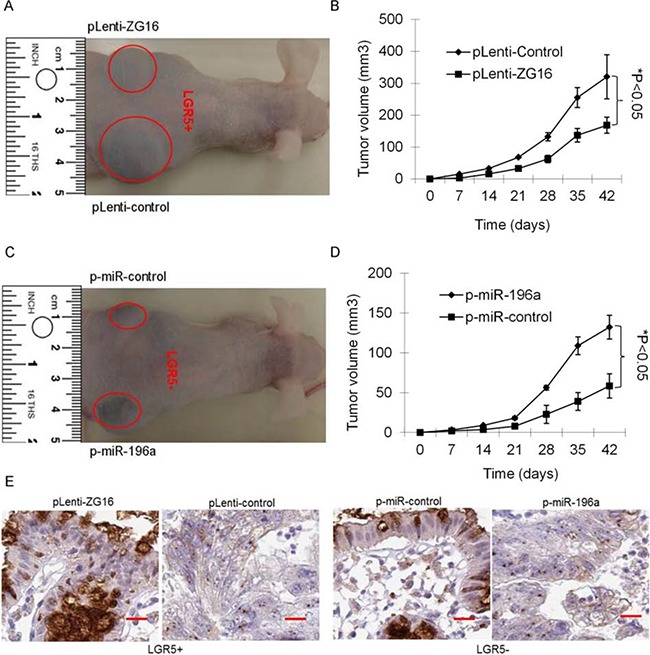
miR-196a/ZG16 contributes tumor growth of stem-like CRCs *in vivo* **A** and **B**. The representative images and tumor volume of tumors that formed from pLenti-ZG16 stably transfected LGR5+ CRC cells grew significantly slower than those formed from pLenti-control transfected LGR5+ CRC cells. **C** and **D**. The representative images and tumor volume of tumors that formed from p-miR-196a stably transfected LGR5- CRC cells grew significantly faster than those formed from p-miR-control transfected LGR5- CRC cells. **E**. The representative images of immunohistochemical (IHC) staining of tumour tissues showed that the expression of ZG16 was significantly increased in the pLenti-ZG16 stably transfected LGR5+ CRC tumors, while significantly decreased in p-miR-196a stably transfected LGR5- CRC tumours.

The effect of stably overexpressing of ZG16 or miR-196a on the tumorigenicity of CRC cells was further investigated in the mouse model *in vivo*. Immunodeficient Balb/C mice were subcutaneously injected with LGR5+ CRC cells stably transfected with pLenti-ZG16 or pLenti-control or LGR5- CRC cells stably transfected with p-miR-196a or p-miR-control. Throughout the tumorigenic period, the tumours that generated from pLenti-ZG16 stably transfected LGR5+ CRC cells grew significantly slower than those generated from pLenti-control transfected LGR5+ CRC cells (Figure 6A and 6B). Meanwhile, the tumours that generated from p-miR-196a stably transfected LGR5- CRC cells grew significantly faster than those from p-miR-control transfected LGR5- CRC cells (Figure 6C and 6D). After 42 days, immunohistochemical (IHC) staining of tumour tissues indicated that ZG16 expression was significantly increased in the pLenti-ZG16 stably transfected LGR5+ CRC tumors, while significantly decreased in p-miR-196a stably transfected LGR5- CRC tumours (Figure 6E). These data suggest that miR-196a/ZG16 contributes to tumor growth of stem-like CRCs *in vivo*.

## DISCUSSION

ZG16 (Zymogen Granule Protein 16) has been identified as a linker molecule in 1998 in the pancreatic acinar cells [7]. It is firstly reported as a secreted protein in human liver, but it has been shown down-regulated in liver cancer [8]. Immunostaining analysis of ZG16 expression showed that it is also located at both the goblet cells and the mucus. ZG16 was also shown highly expressed in colon [9]. ZG16 expression was specifically observed in the mucus-secreting cells of the digestive system by immunohistochemical staining [10]. However, the expression and role of ZG16 in CRCs have not been reported. Here, we found that ZG16 is significantly decreased in CRC in TCGA dataset and our validating dataset. The loss of ZG16 showed significantly correlated with poor prognosis of CRC. We also found that the expression of ZG16 highly correlates with CRC related genes which were regulated by the APC/CTNNB1 pathway, suggesting that loss of ZG16 may also play an important role in CRC.

It is well known that both APC and CTNNB1 mutation are frequently happened in CRCs and are critical for progression and metastasis of CRCs [5]. We further investigated the potential correlation of ZG16 with cancer stem cell markers of CRC. Interestingly, we observed that loss of ZG16 was highly correlated with overexpression of LGR5, which has been reported as a marker for stem-like cells in CRC. The CRC stem cells were first identified on the basis of surface marker CD133 expression. Markers that have been described to characterize CRC stem cells include EphB2^high^, EpCAM^high^/CD44^+^/CD166^+^, ALDH^+^, LGR5^+^, CD44v6^+^ and DCLK1 [11]. To further validate this, we isolated LGR+ and LGR5- subpopulation cells from CRC cells using flow cytometry cell sorting. Interestingly, we found that ZG16 was significantly down-regulated in LGR+ CRC cell population. Furthermore, we found that overexpression of ZG16 inhibits growth and sphere formation of stem-like CRC cells, suggesting that loss of ZG16 may also play an important role in CRC initiation and stemness.

Since the critical role of ZG16 in CRC cell growth and tumor initiation, it is important to understand the upstream regulation of ZG16 in CRC. microRNAs have been predicted to regulate at least one-third of human genesand the regulation by miRNA is pervasive in animals [12]. To identify the potential regulatory microRNAs of ZG16, we used bioinformatics prediction and experiment validation like luciferase reporter assay and western blotting assay. Finally, we identified that miR-196a is an upstream regulator of ZG16 in CRC cells. Previous study showed that miR-196a has the pro-oncogenic role in CRCs. Overexpression of miR-196a has been shown to promote migration, invasion and chemotherapy sensitivity of tumor cells [13]. Moreover, miR-196a can promote lung metastases by tail vein injection in mice [13]. The overexpression of miR-196a has also been shown to play important role in other cancer types. For instance, circulating miR-196a/b has been reported as the novel biomarkers associated with metastatic gastric cancer [14]. High miR-196a has been shown to to be associated with poor prognosis of high-grade glioma [15]. it's the oncogenic role of miR-196a in glioblastoma multiforme has been shown to inhibit IκBα[16]. MiR-196a has also been shown to promote the progression of pancreatic cancer by targeting NFKBIA [17], which may also related to downregulation of ZG16 in pancreatic cancer. Here, we found that miR-196a promotes cell growth and stemness of CRC cells and miR-196a/ZG16 contributes tumor growth of stem-like CRCs *in vivo*. In conclusion, this study demonstrated that loss of ZG16 is regulated by miR-196a and contributes to stemness and progression of colorectal cancer, which may provide a promising therapeutic strategy for advanced CRCs.

## MATERIALS AND METHODS

### Cell culture and tissues

Human CRC cell line HT29 was bought from American Type Culture Collection (ATCC) and cultured in McCoy's 5a Medium (Thermo Fisher Scientific) with 10% FBS and 100 U/mL penicillin/streptomycin (Sigma, St Louis, MO). The LGR5+ and LGR5- HT29 cells were sorted from parental cells using flow cytometry. The CRC tissue samples were collected and proved by the Hospital Committees for Ethical Review of Research Involving Human Subjects.

### Real-Time Quantitative Reverse Transcription PCR (qRT-PCR)

Total RNA was extracted using RNeasy Mini Kit (Qiagen, Hilden, Germany). The concentration of isolated total RNA was measured by NanoDrop ND-1000 Spectrophotometer (Agilent, CA). The qRT-PCR was performed using SsoFast™ EvaGreen® Supermix (Bio-Rad) with hypoxanthine phosphoribosyltransferase 1 (HPRT1) as an internal control, as described previously [18] and Supplementary Materials and Methods.

### Animal studies

The nude mice were 5-6 weeks old and 20g in weight. They were bred in aseptic conditions and kept at a constant humidity and temperature (25-28°C) according to standard guidelines under a protocol approved by Hospital Committees for Ethical Review of Research Involving Animals. All mice were injected subcutaneously with 100 ul suspension (5 × 10^6^) of LGR5+ cells with stable transfection of pLenti-ZG16 or pLenti-control, or LGR5- cells with stable transfection of p-miR-196a or p-miR-control. The size of the tumor was measured once a week with calipers, and the volume of tumor was determined using the simplified formula of a rotational ellipsoid (length × width^2^ ×0.5).

The rest of materials and methods are provided in the online supplementary data.

## SUPPLEMENTARY TABLE




