# Supplementary material for: Loss of ZG16 is regulated by miR-196a and contributes to stemness and progression of colorectal cancer
Source: Oncotarget. 2016 Nov 17;7(52):86695–703. doi: 10.18632/oncotarget.13435 (PMC5349946; doi:10.18632/oncotarget.13435)
Supplement: Supplementary file 1 [file oncotarget-07-86695-s001.pdf]

Loss of ZG16 is regulated by miR-196a and contributes to stemness and progression of colorectal cancer

SUPPLEMENTARY MATERIALS AND METHODS

Real-Time quantitative reverse transcription PCR (qRT-PCR)

Total RNA was extracted using RNeasy Mini Kit (Qiagen, Hilden, Germany). The concentration of isolated total RNA was measured by NanoDrop ND-1000 Spectrophotometer (Agilent, CA). For miRNA detection, the total RNA samples were polyadenylated and reverse transcribed using the NCode™ VILO™ miRNA cDNA Synthesis Kit and EXPRESS SYBR® GreenER™ miRNA qRT-PCR Kits (Invitrogen, CA) according to the manufacturer's instructions. The U6 snRNA was used as the internal control. For mRNA detection, the total RNA was reversely transcribed by using SuperScript III First-Strand Synthesis System for RT-PCR (Invitrogen, CA). The qPCR were performed by using SsoFast™ EvaGreenH Supermix (Bio-Rad). The HPRT1 was used as the internal control.

MTS assays

The LGR5+ or LGR5- cells were seeded into 96-well plates at a density of 5×10^3 per well (100 µl) and stable transfected with pLeni-ZG16 or p-miR-196a. For the MTS assay, the CellTiter 96® AQueous One Solution Cell Proliferation Assay kit (Promega, Madison, WI, USA) was used following the manufacturer's instruction. Briefly, at 2 h before each of the desired time points (24 h, 48 h, 72 h and 96 h), 20 µl of the MTS reagent was added into each well and cells were incubated at 37°C for around 2 h. The absorbance was detected at 490 nm using a Wallac Victor 1420 Multilabel plate reader. All the experiment was repeated three times.

Sphere formation assay

Single stable transfected LGR5+ or LGR5- cells (1×10^3) were plated onto a 24-well ultra-low attachment plate (Corning, Corning, NY) in serum-free DMEM-F12, supplemented with 2 mM L-glutamine, 1% sodium pyruvate, 1% MEM nonessential amino acids, 100 µg/ml penicillin, and 100 U/ml streptomycin supplemented with 20 ng/ml epithelial growth factor (EGF) and 10 ng/ml fibroblast growth factor-2 (bFGF) (Invitrogen). After 2-3 weeks of culture, the number of spheres (diameter > 40 µm) was manually counted in three randomly selected fields at a magnification of 40× under an inverted fluorescence microscope. This assay was performed in triplicate in three independent experiments.

Flow cytometry

The stably transfected LGR5+ or LGR5- cells were resuspended in 100µl staining buffer containing 10% FBS and put on ice for 20 min to block Fc receptors. After incubating with primary biotin rat anti-human Lgr5 (N-Terminal) or isotype control (BD Biosciences, Bedford, MA) for 1-2h on ice in the dark, the cells were then washed with 1ml ice-cold staining buffer for two times and centrifuged (400g) at 4oC for 5 min. The collected cells were suspended in 500µl staining buffer solution and were evaluated using BD FACSAria™ III sorter (BD Biosciences).

Luciferase reporter assay

For the reporter assay, HEK293 cells were plated onto 24-well plates and transfected with the constructs (pGL3-ZG16-wt or pGL3-ZG16-mut) and p-miR-196a or p-miR-control vectors using the Lipofectamine® 3000 reagent (Thermo Fisher Scientific). A Renilla luciferase vector pRL-SV50 (Promega, Madison, WI) was co-transfected to normalize the difference in the transfection efficiency. After 48h, the cells were harvested and assayed using the dual-luciferase reporter assay system (Promega, Madison, WI) according to the manufacturer's instructions. Results were obtained from three independent experiments performed in duplicate.

Western blotting

Protein was isolated using RIPA and their concentrations determined using the Bradford protein assay (Bio-Rad, Hercules, CA, USA). Antibodies for Western blot were: rabbit anti-secretory lectin ZG16 antibody (Abcam, Cambridge, UK) and goat GAPDH (GenScript, NJ).

Immunohistochemistry (IHC)

The paraffin-embedded tissue samples from animal studies were cut in 5-µm sections and placed on polylysine coated slides; then the samples were deparaffinized in xylene and rehydrated using a series of graded alcohols. Antigen retrieval was performed by heat mediation in citrate buffer (pH 6) (Dako). Samples were blocked with 10% goat serum before incubating

with primary antibody. The samples were incubated overnight using a primary antibody, Anti-ZG16 (Abcam, Cambridge, UK) (1:100) or an isotype-matched IgG as a negative control in a humidified container at 4°C. The intensity of staining was evaluated on the scale of 0 to 4 according to the percentage of positive tumors (0, negative control; 1, 0%-10%; 2, 10%-25%; 3, 25%-50% and 4, >50%).

Survival and statistical analysis

The experimental data are presented as the mean ± standard deviation (SD). All statistical analyses were performed using ANOVA or a two-tailed Student's t test (GraphPad Prism 5). The survival curves were created using the Kaplan-Meier method and statistically compared using a log-rank test. Differences were considered statistically significant when the P-value was less than 0.05.

SUPPLEMENTARY TABLE

Supplementary Table S1: ZG16 highly correlated genes.

Supplementary File 1
