# Supplementary material for: Loss of ZG16 is regulated by miR-196a and contributes to stemness and progression of colorectal cancer
Source: Oncotarget. 2016 Nov 17;7(52):86695–703. doi: 10.18632/oncotarget.13435 (PMC5349946; doi:10.18632/oncotarget.13435)
Supplement: Supplementary file 2 [file oncotarget-07-86695-s002.docx]

**Table S1. ZG16 highly correlated genes**

| Probeset ID | r | Gene Symbol | p-value  (T vs. N) | Fold-Change  (T vs. N) |
| --- | --- | --- | --- | --- |
| 220026_at | 0.961153 | CLCA4 | 5.63E-13 | -130.873 |
| 205950_s_at | 0.937526 | CA1 | 2.21E-23 | -170.326 |
| 206209_s_at | 0.932357 | CA4 | 1.12E-15 | -117.217 |
| 206208_at | 0.927702 | CA4 | 1.96E-17 | -80.7864 |
| 207003_at | 0.922197 | GUCA2A | 1.85E-16 | -126.837 |
| 220834_at | 0.91526 | MS4A12 | 7.14E-17 | -149.393 |
| 219669_at | 0.90698 | CD177 | 3.28E-19 | -91.7373 |
| 203908_at | 0.898257 | SLC4A4 | 5.26E-21 | -131.658 |
| 206198_s_at | 0.889005 | CEACAM7 | 2.24E-10 | -57.9897 |
| 208399_s_at | 0.879639 | EDN3 | 2.79E-20 | -22.7777 |
| 209301_at | 0.879497 | CA2 | 2.13E-12 | -48.6439 |
| 204673_at | 0.879408 | MUC2 | 3.70E-05 | -15.7212 |
| 219799_s_at | 0.877763 | DHRS9 | 1.97E-11 | -24.8679 |
| 211848_s_at | 0.877554 | CEACAM7 | 1.80E-08 | -58.2302 |
| 205259_at | 0.876543 | NR3C2 | 1.08E-14 | -21.8364 |
| 214598_at | 0.867274 | CLDN8 | 5.27E-16 | -73.8136 |
| 217109_at | 0.86644 | MUC4 | 1.55E-08 | -11.0834 |
| 207502_at | 0.862934 | GUCA2B | 6.48E-25 | -84.7085 |
| 217207_s_at | 0.860585 | BTNL3 | 1.07E-16 | -6.68937 |
| 212814_at | 0.86 | AHCYL2 | 4.43E-20 | -13.1227 |
| 210107_at | 0.858423 | CLCA1 | 2.74E-05 | -11.7324 |
| 215100_at | 0.85788 | ADTRP | 3.07E-18 | -5.18555 |
| 210739_x_at | 0.857102 | SLC4A4 | 6.88E-32 | -23.7205 |
| 203240_at | 0.85293 | FCGBP | 3.63E-06 | -25.6175 |
| 209875_s_at | -0.85289 | SPP1 | 1.58E-08 | 15.3508 |
| 215672_s_at | 0.851511 | AHCYL2 | 1.87E-19 | -22.5092 |
| 201739_at | 0.850999 | SGK1 | 4.19E-14 | -8.06769 |
| 209791_at | 0.849406 | PADI2 | 6.36E-20 | -20.3583 |
| 205464_at | 0.84662 | SCNN1B | 2.02E-17 | -40.4087 |
| 205593_s_at | 0.842146 | PDE9A | 1.54E-15 | -36.6123 |
| 207222_at | 0.841233 | LOC100652777 /// PLA2G10 | 5.55E-11 | -5.52431 |
| 213738_s_at | 0.836171 | ATP5A1 | 2.94E-11 | -1.90551 |
| 213905_x_at | -0.83608 | BGN | 3.66E-11 | 12.8783 |
| 211494_s_at | 0.835669 | SLC4A4 | 7.75E-26 | -34.1104 |
| 201666_at | -0.83167 | TIMP1 | 5.89E-16 | 7.44294 |
| 205185_at | 0.831449 | SPINK5 | 1.23E-08 | -11.8481 |
| 214696_at | 0.831239 | MIR22 /// MIR22HG | 1.05E-17 | -11.4679 |
| 204368_at | 0.829879 | SLCO2A1 | 5.02E-10 | -5.74653 |
| 204378_at | 0.825717 | BCAS1 | 2.99E-14 | -6.10768 |
| 207504_at | 0.825593 | CA7 | 1.45E-22 | -51.87 |
| 214433_s_at | 0.824362 | SELENBP1 | 8.83E-12 | -9.47413 |
| 209735_at | 0.822661 | ABCG2 | 1.84E-32 | -137.715 |
| 210738_s_at | 0.822446 | SLC4A4 | 5.86E-23 | -26.6677 |
| 213407_at | 0.820605 | PHLPP2 | 2.24E-19 | -7.64255 |
| 210512_s_at | -0.82036 | VEGFA | 2.77E-11 | 2.95439 |
| 217110_s_at | 0.820008 | MUC4 | 6.15E-12 | -17.9632 |
| 212593_s_at | 0.819187 | MIR4680 /// PDCD4 | 1.96E-18 | -3.4126 |
| 202242_at | 0.817593 | TSPAN7 | 6.73E-14 | -19.3369 |
| 207432_at | 0.81641 | BEST2 | 7.04E-18 | -5.70255 |
| 207601_at | 0.816122 | SULT1B1 | 2.45E-08 | -6.35069 |
| 201261_x_at | -0.81504 | BGN | 1.01E-10 | 8.06239 |
| 205945_at | 0.814928 | IL6R | 2.96E-22 | -15.0654 |
| 217845_x_at | 0.814528 | HIGD1A | 7.33E-14 | -2.93253 |
| 206784_at | 0.813904 | AQP8 | 1.64E-25 | -639.831 |
| 206100_at | 0.812509 | CPM | 2.77E-18 | -18.9061 |
| 206561_s_at | 0.810915 | AKR1B10 | 6.71E-12 | -47.9603 |
| 205480_s_at | 0.809269 | UGP2 | 1.58E-21 | -4.03738 |
| 208780_x_at | 0.808559 | VAPA | 6.64E-11 | -1.93674 |
| 221896_s_at | 0.808509 | HIGD1A | 7.02E-14 | -3.53005 |
| 220180_at | 0.808086 | CCDC68 | 6.77E-14 | -18.0405 |
| 221305_s_at | 0.80762 | UGT1A8 /// UGT1A9 | 5.23E-21 | -29.1614 |
| 205861_at | 0.805961 | SPIB | 1.20E-20 | -28.9251 |
| 209373_at | 0.80482 | MALL | 1.27E-11 | -6.34056 |
| 213624_at | 0.804011 | SMPDL3A | 5.36E-16 | -12.7887 |
| 220266_s_at | 0.801734 | KLF4 | 1.99E-11 | -17.0839 |
| 205097_at | 0.801571 | SLC26A2 | 1.37E-10 | -60.2101 |
| 221004_s_at | 0.800954 | ITM2C | 5.91E-09 | -5.65845 |
